# Infection prediction in swine populations with machine learning

**DOI:** 10.1038/s41598-023-43472-5

**Published:** 2023-10-18

**Authors:** Avishai Halev, Beatriz Martínez-López, Maria Clavijo, Carlos Gonzalez-Crespo, Jeonghoon Kim, Chao Huang, Seth Krantz, Rebecca Robbins, Xin Liu

**Affiliations:** 1 Department of Mathematics, University of California, Davis, Davis, CA USA; 2 Department of Medicine and Epidemiology, Center for Animal Disease Modeling and Surveillance (CADMS), School of Veterinary Medicine, University of California, Davis, Davis, CA USA; 3 Department of Veterinary Diagnostic & Production Animal Medicine (VDPAM), Iowa State University, Ames, IA USA; 4 Department of Computer Science, University of California, Davis, Davis, CA USA; 5 Tosh Farms, Henry, TN USA; 6 Pig Improvement Company, Hendersonville, TN USA

**Keywords:** Diseases, Influenza virus, Viral infection, Risk factors, Applied mathematics, Computer science, Scientific data

## Abstract

The pork industry is an essential part of the global food system, providing a significant source of protein for people around the world. A major factor restraining productivity and compromising animal wellbeing in the pork industry is disease outbreaks in pigs throughout the production process: widespread outbreaks can lead to losses as high as 10% of the U.S. pig population in extreme years. In this study, we present a machine learning model to predict the emergence of infection in swine production systems throughout the production process on a daily basis, a potential precursor to outbreaks whose detection is vital for disease prevention and mitigation. We determine features that provide the most value in predicting infection, which include nearby farm density, historical test rates, piglet inventory, feed consumption during the gestation period, and wind speed and direction. We utilize these features to produce a generalizable machine learning model, evaluate the model’s ability to predict outbreaks both seven and 30 days in advance, allowing for early warning of disease infection, and evaluate our model on two swine production systems and analyze the effects of data availability and data granularity in the context of our two swine systems with different volumes of data. Our results demonstrate good ability to predict infection in both systems with a balanced accuracy of $85.3\%$ on any disease in the first system and balanced accuracies (average prediction accuracy on positive and negative samples) of $58.5\%$, $58.7\%$, $72.8\%$ and $74.8\%$ on porcine reproductive and respiratory syndrome, porcine epidemic diarrhea virus, influenza A virus, and *Mycoplasma hyopneumoniae* in the second system, respectively, using the six most important predictors in all cases. These models provide daily infection probabilities that can be used by veterinarians and other stakeholders as a benchmark to more timely support preventive and control strategies on farms.

## Introduction

The United States is the world’s second largest producer of pork, producing 12.6 million metric tons of pork in 2021—over 11% of total global production^1^. In 2021, the industry generated over $24 billion in revenue and employed over 600 thousand people at various stages in the production process^2^. In the United States, hog producers generally fall into one of three categories. In recent decades, the hog industry has moved away from farrow-to-finish operations, which raise hogs from birth to slaughter, and specialized into multi-site operations with separate nurseries, finishers, and sow sites in order to maximize farm efficiency and lower production costs^3^. In doing so, individual farms have grown larger and consolidated, with the total number of farms declining over 70% since 1990^3^.

This shift, however, necessitates the movement of large numbers of pigs between sites, allowing for increased numbers of contact points and increasing the chances of diseases propagating between farms. This trend toward multi-site operations, in addition to the increasing globalization of the swine industry, has led to an increase in the vulnerability of the swine industry to both endemic and emerging pathogens. For example, in 2013, the emergence of the porcine epidemic diarrhea virus in the United States led to the loss of over 10% of the domestic pig population^4,5^.

Current approaches implemented in swine production systems for disease control are labor and capital intensive, including strict biosecurity regimens, disease surveillance, and vaccine administration^6^. Consequently, the swine industry has become interested in the implementation of disease forecasting tools in swine populations to strategically use resources and available fixed infrastructure^7^. The ability to do so hinges on accurate identification of disease transmission pathways. Disease propagation is caused by both direct and indirect contacts, with the transportation of infected pigs as well as the airborne movement of disease being significant sources of outbreaks^8–10^. Outbreaks are sudden rises in the number of cases in a disease; as a result, they are always preceded by disease infections, which we study here. Infections can lead to outbreaks if left undetected, untreated, or uncontrolled; infection detection, however, allows for early mitigation and treatment preparation.

Traditionally, the most common epidemiological models for disease prediction have been compartmental models, which are knowledge-driven mechanistic models. The most common models include the SIR—Susceptible, Infectious, and Recovered—and the SEIR—Susceptible, Exposed, Infectious and Recovered models^11,12^. Both SIR and SEIR models are reasonably effective at predicting outbreaks in the short-term; they tend to struggle, however, with more complicated dynamics and with early prediction^13–15^.

Another approach to disease prediction is agent-based modeling, in which models simulate the actions and interactions of individual agents, each acting autonomously under a set of assumptions. These models have been shown to be more accurate to compartmental models in predicting spread of different diseases^16,17^. However, they are computationally intensive, can be difficult to parameterize and calibrate, and commonly-made assumptions have been criticized as being unrealistic^18,19^.

Machine learning (ML) has emerged as a powerful tool in disease prediction. In machine learning, data is leveraged to uncover insights about a task of interest by using trends from past data to generalize and infer on new data. Machine learning has had success in a variety of applications, including computer vision, natural language processing, speech recognition, fraud detection, and robotic locomotion, and has recently been applied effectively in public health^20–25^.

In the swine field, Liang et. al wielded outbreak and meteorological data to predict outbreaks of African swine fever (ASF) on a global scale with ML^26^. The authors trained ML models on their collected data and found that random forest models were effective in ASF outbreak prediction. Shamsabardeh et al. predicted porcine reproductive and respiratory syndrome (PRRS) outbreaks using machine learning^27^. Their model leveraged internal production and movement data as well as external factors to predict disease within the production period and achieved a high degree of accuracy. However, the model suffers from temporal granularity issues, where outbreaks can only be predicted at the time-scale of a production cycle, which range from three to over six months.

Silva et al. used machine learning methods to determine biosecurity and production factors that impacted outbreaks of PRRS in swine farms^28^. They focus on two variable selection strategies and find subsets of the overall feature set that outperform naïvely using the entire feature set in prediction. Machado et al. utilized movements of animals between farms and neighborhood attributes to predict outbreaks of PEDV in sow farms in one-week periods^29^. The authors used neighborhood attributes such as hog density, environmental data such as vegetation, wind speed, temperature, precipitation, and topographical features such as slope to capture local area spread of disease. Combined with long-distance movements, their model was able to predict outbreaks of PEDV on sow farms with greater than 80% accuracy on data collected over the course of one year. In Paploski et al.  the authors used XGBoost (gradient boosting) models to forecast outbreak of PEDV in near real time by leveraging scheduled movement data for the upcoming week as well as five weeks of historical data to predict outbreaks^7^.

In our study, we design a model to predict infection using three disease pathways: direct contact, indirect contact, and local area spread. We consider data from two farm systems, one with low data granularity and one with high data granularity, and show that we can build models that adapt to cases with sparse data, where we infer global disease trends on farms, and cases with granular data, where we are able to predict case trends for specific diseases: porcine reproductive and respiratory syndrome (PRRS), porcine epidemic diarrhea virus (PEDV), influenza A virus (IAV), and *Mycoplasma hyopneumoniae* (MHP). We access diagnostic and movement data collected daily and production data collected weekly on sow and nursery/finishing farms over a period of multiple years and leverage it to predict infection with a daily time granularity. We analyze a wide variety of machine learning models, including support vector machines, tree-based models such as decision trees and random forests, and neural networks, and show that models are able to predict infection with seven and 30 day advance warning. The results of this study are also useful to detect abnormal diagnostic results for further scrutiny, helping to detect diseases earlier and more rapidly and aiding practitioners in effectively controlling outbreaks. We also propose additional detection methods when more complete data is available.

## Results

**Figure 1:**
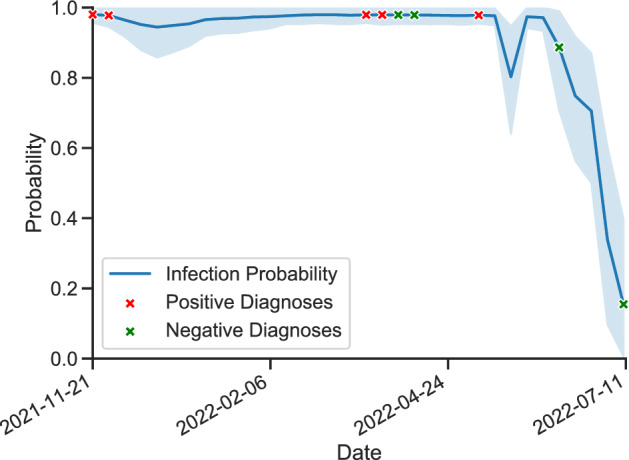
IAV infection prediction on a single farm using our model compared to true diagnoses. Outbreak probabilities are evaluated on a weekly basis.

We trained and evaluated a variety of machine learning models and dimension reductions and showed that the models are able to effectively predict infections, defined as a positive test result on a specific date, using data collected over a period of time ending on that date. Due to imbalances in the number of positive and negative samples, we use balanced accuracy scores to evaluate our model, which is the weighted accuracy on both classes. The best performance is provided with a 60-day window: on system A, the top-performing model—a random forest with 10 estimators—has a cross-validated balanced accuracy of $0.897\pm 0.05$ and provides a balanced accuracy of 0.853 on the test set, using the six most important features. On system B, model performance lags slightly behind—especially in generalizing to the test set. Our top-performing model, an auto-encoder neural network model, scores $0.633 \pm 0.05$, $0.788 \pm 0.08$, $0.777 \pm 0.06$, and $0.906 \pm 0.05$ in cross-validation on PRRS, PEDV, IAV, and MHP, respectively, and achieves balanced accuracies of 0.585, 0.587, 0.728 and 0.748 on the test sets of PRRS, PEDV, IAV, and MHP, respectively, using the six most important features. Note that cross-validation scores, not test scores, are used to determine the best performing model; this is to avoid using and thus leaking any information from the test set in the model and window selection process. An example of our model being used for inference of IAV infection on a specific farm can be seen in Fig. 1.

### Infection prediction in system A

We consider diagnoses collected on 60 farms in system A, of which 40 are finishing sites, 10 are sow sites, and 10 do not contain production data. There are approximately 1000 total samples, with the exact number varying slightly with the window length; of these, roughly 11% are positive samples (see the “Methods” section for exact counts).

The best-performing models across all window lengths are ensemble tree models: random forests and gradient boosting, with cross-validation balanced accuracies of 0.918 and 0.898 with data collected over a 60-day historical window, respectively. Random forests excel yet slightly overtrain compared to gradient boosting, which often overtake them in performance on the test set: the corresponding random forest and gradient boosting models with 10 estimators obtain test accuracies of 0.789 and 0.792 for random forests and gradient boosting, respectively. Second, while random forests and gradient boosting models provide similar performance, the optimal thresholds for random forest are higher than those for gradient boosting, suggesting that the random forest model is able to better differentiate positive and negative samples in the probability space; this effect is particularly pronounced in models trained on data collected over 30- and 90-day historical windows. In other words, the random forest model gives positive samples higher probabilities of being positive than the gradient boosting model. Details on these models and additional models are available in Table 1.

**Table 1:** Balanced accuracy with selected hyperparameters on system A. Balanced accuracy represents the average recall, weighted between positive and negative samples. Columns *CV* and *Test* correspond to balanced accuracy scores in cross-validation and on the test set, respectively, while *Thresh* gives the optimal threshold for that model as determined via the metric computation process. Higher thresholds imply better model discrimination between positive and negative predictions. Results with additional models and hyperparameters are available in Supplementary Table S1.

	Trees	14 Day			30 Day		
		CV	Test	Thresh	CV	Test	Thresh
Random forest	10	0.895 ± 0.040	0.829	0.095	0.879 ± 0.033	0.833	0.160
	25	0.892 ± 0.037	0.878	0.190	0.873 ± 0.027	0.828	0.198
	100	0.885 ± 0.047	0.842	0.204	0.880 ± 0.030	0.824	0.194
Gradient boosting	10	0.890 ± 0.020	0.849	0.064	0.891 ± 0.025	0.801	0.053
	25	0.880 ± 0.021	0.844	0.109	0.891 ± 0.025	0.792	0.060
	100	0.888 ± 0.012	0.840	0.073	0.889 ± 0.028	0.801	0.064

		60 Day			90 Day		
Random forest	10	0.918 ± 0.022	0.789	0.212	0.898 ± 0.025	0.882	0.216
	25	0.915 ± 0.028	0.825	0.164	0.898 ± 0.022	0.896	0.218
	100	0.913 ± 0.026	0.792	0.212	0.894 ± 0.026	0.891	0.186
Gradient boosting	10	0.898 ± 0.028	0.792	0.140	0.872 ± 0.036	0.856	0.051
	25	0.898 ± 0.028	0.758	0.141	0.860 ± 0.038	0.868	0.062
	100	0.897 ± 0.030	0.769	0.132	0.865 ± 0.034	0.887	0.102

In addition, the threshold-determining process is rendered irrelevant on decision tree models as they predict binary probabilities for all samples. Specifically, since the decision tree models are complete and all leaves are pure, predicted probabilities are either zero or one; as a result, threshold selection loses its meaning.

We see that the model is relatively robust to dimensionality reduction with some slight variation by performing a sensitivity analysis on the dimension reduction parameter, with performance remaining relatively similar across different numbers of PCA components. This suggests linear dependence and a lack of predictive power of some features. In general, the stratified dimension reduction outperforms the unstratified dimension reduction on system A, with ten close out (Finishing Farm key performance indicator) and ten sow features providing consistently high performance. Detailed results of this analysis are available in Supplementary Discussion 2.1.

#### Important predictors

**Figure 2:**
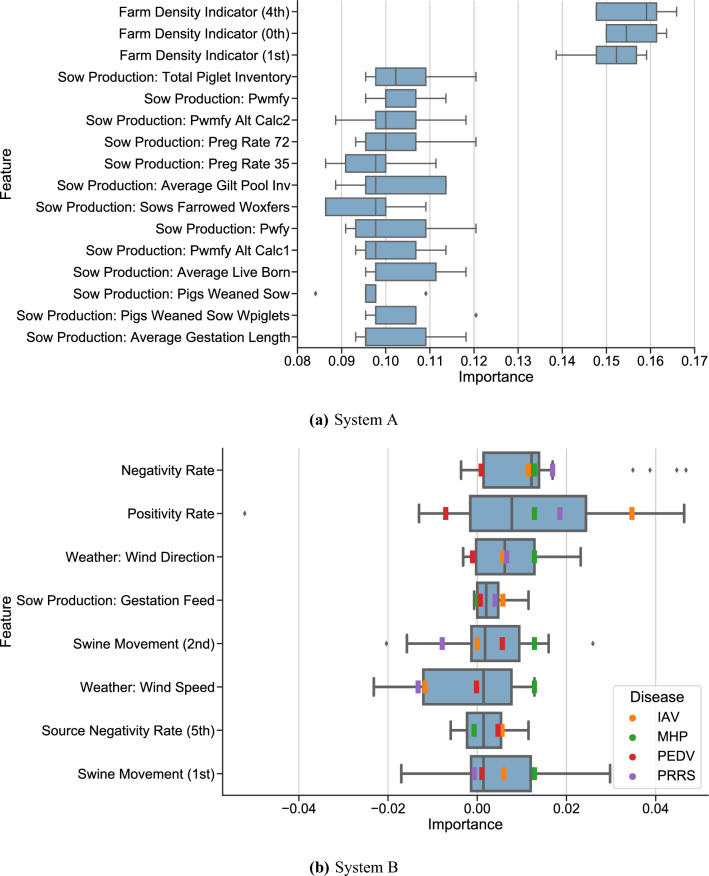
Permutation importance of both systems; features are ordered by median score. Medians of importance with respect to models trained on each disease displayed for system B. Feature descriptions as well as a full enumeration of important features by disease in system B are available in Supplementary Tables S11 and S12, respectively. Feature names in the figure follow the form $\langle$Feature Name (Source Farm Number)$\rangle$. For example, Swine Movement (1st) denotes the feature representing the number of incoming swine from the first source farm within the historical window. Source farms are zero-indexed. Farm Density Indicators (*n*th) give the distance of the *n*th nearest farm, providing information about local farm density. See Supplementary Methods 3.2 for additional details on naming conventions.

**Figure 3:**
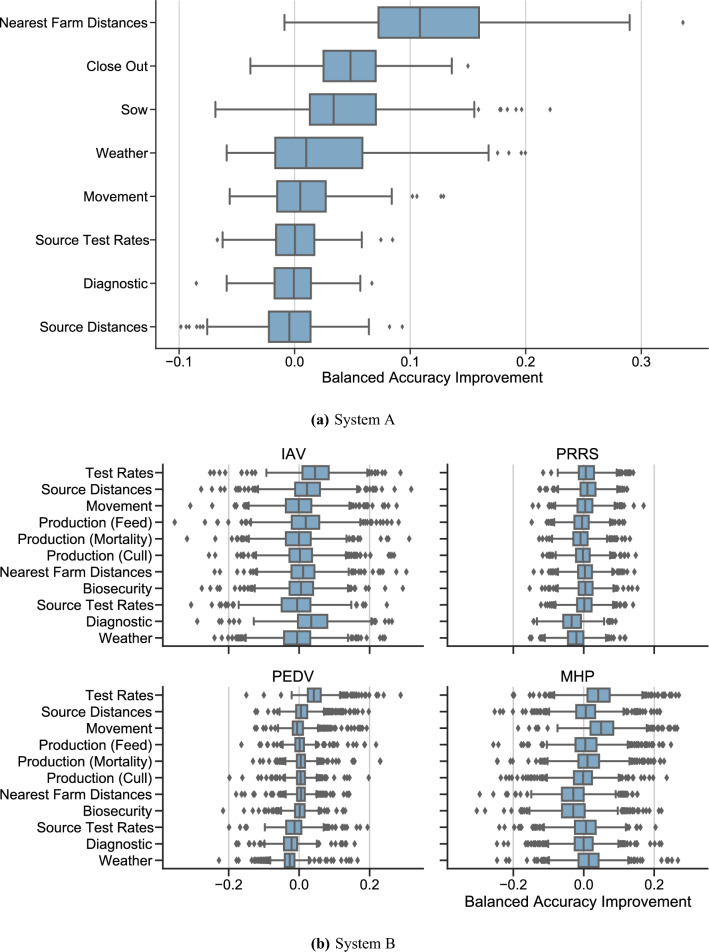
Group feature importance. Each point represents an increase in balanced accuracy from adding one group of features to a model using other groups of features.

We find that the distance of the five closest neighboring farms—a proxy for local farm density—and sow production features concerning piglet production rates are the most significant features. Specifically, total piglet inventory, the number of pigs weaned per mated female per year (PWMFY), pregnancy rates and average gilt pool inventory (total gilt days per days in period) are all particularly valuable to the model. The importances of these and additional important features can be seen in Figs. 2a and 3a.

By performing a marginal feature importance, where we consider the value of adding a group of features as a whole to a model, we find that the distance of nearby farms are again important, as are data from close out reports (from finishing farms) and sow production data. These sets of features beget accuracy increases of 12.5, 4.9 and 4.9 percentage points, respectively. As we group together the entire sets of each type of production data, this analysis suggests that on a high level, close out data and sow production data add value to the model: while it does not provide specific insight on which production features provide this value, we can infer from out previous permutation feature importance that total piglet inventory, PWMFY, pregnancy rates and average gilt pool inventory provide this value for sow production, for example. With the exception of source test rates, all sets of features have a positive effect on the median model, albeit with significant variation in the magnitude of these effects.

#### Performance with selected important predictors

**Figure 4:**
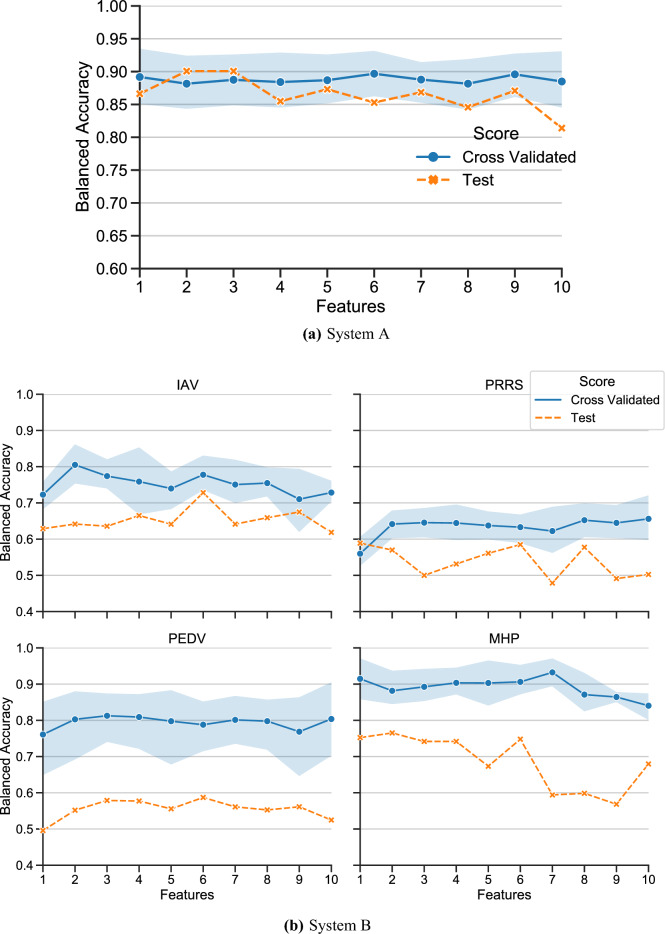
Model performance with feature selection by permutation feature importance using ten most important features. Each point represents the model’s performance with the *n* best features for that disease/system. Results with all features available in Supplementary Figure S4.

Based on the feature importances from the previous section, we select the *n* most important features and pass them to the final steps in the model. Cross-validated and test set scores are presented in Fig. 4a. From this figure, it is apparent the model performs best with the top six features, peaking with a cross validated balanced accuracy of $0.897\pm 0.04$ and a corresponding test accuracy of 0.853. Past this critical point, performance improvements taper off and eventually mildly decrease. In system A, while cross-validated scores remain effectively flat as less significant features are added, test scores gradually decrease, suggesting mild overfitting. However, this effect is mild, and may be due to randomness.

#### Early prediction

Our results show that longer data collection windows generally performed better than shorter windows, with models trained on 90-day collection windows achieving higher accuracies than models trained on 14- and 30-day windows and models trained on 60-day collection windows outperforming all three (Table 1). We would thus expect the model to leverage information not only from the immediate past but from the more distant past as well. We train the top-line model—random forests with 10-feature stratified PCA on these new datasets to determine our ability to forecast into the future and achieve cross-validated balanced accuracy scores of 0.883 and 0.891 for 7- and 30-day early prediction utilizing data collected over a 60-day historical window, respectively, meaning we predict positive tests 7- and 30-days in the future with data collected over the previous 60 days. This shows that the model does not suffer when performing early prediction (Supplementary Figure S3).

### Infection prediction in system B

In system B, we focus on diagnoses collected on 148 farms, of which 132 are nursing/finishing (NurFin) sites, 15 are sow sites and 1 is a boar site, which we group together with the sow sites. With data collected over 60-day historical windows, we have 3215, 3366, 681, and 343 total samples for PRRS, PEDV, IAV and MHP, respectively, of which 708, 475, 271 and 97 are positive, respectively.

The models with the highest accuracy on system B differ from those on system A (Table 2). The best model is the auto-encoder MLP model, which provides the most consistent results across all diseases and best generalizes to validation and test sets. By extension, other models tested, such as Random Forests and Gradient Boosting, overfit on noisy signals present in the training data, leading to high training set scores but lower cross-validation and test set scores (Table 2). This is further compounded by distribution shift between the training and test sets as we discuss in the following section.

**Table 2:** Balanced accuracy with selected hyperparameters on system B. Columns *CV* and *Test* correspond to balanced accuracy scores in cross-validation and on the test set, respectively, while *Thresh* gives the optimal threshold for that model as determined via the metric-computation process. Results with additional models and hyperparameters available in Supplementary Tables S2 and S3.

	PRRS			PEDV		
	CV	Test	Thresh.	CV	Test	Thresh.
MLP						
Hidden layers						
(4, 2, 4)	0.637 ± 0.05	0.575	0.321	0.651 ± 0.14	0.537	0.688
(32, 4, 32)	0.640 ± 0.06	0.523	0.256	0.765 ± 0.09	0.525	0.251
(32, 32)	0.626 ± 0.06	0.487	0.183	0.760 ± 0.13	0.527	0.460
Random forest						
Trees						
5	0.604 ± 0.06	0.618	0.480	0.697 ± 0.10	0.503	0.394
10	0.619 ± 0.06	0.615	0.462	0.720 ± 0.11	0.511	0.354
25	0.629 ± 0.05	0.564	0.468	0.723 ± 0.12	0.553	0.276
Gradient boosting						
Trees						
5	0.635 ± 0.07	0.568	0.357	0.719 ± 0.09	0.567	0.144
10	0.607 ± 0.06	0.614	0.430	0.679 ± 0.07	0.499	0.220
25	0.632 ± 0.07	0.553	0.353	0.723 ± 0.10	0.554	0.164

	IAV			MHP		
MLP						
Hidden layers						
(4, 2, 4)	0.670 ± 0.04	0.637	0.430	0.723 ± 0.13	0.579	0.502
(32, 4, 32)	0.661 ± 0.08	0.710	0.336	0.706 ± 0.16	0.641	0.343
(32, 32)	0.654 ± 0.09	0.623	0.292	0.716 ± 0.16	0.633	0.445
Random forest						
Trees						
5	0.669 ± 0.04	0.540	0.424	0.836 ± 0.07	0.589	0.282
10	0.683 ± 0.05	0.560	0.462	0.82 ± 0.10	0.604	0.254
25	0.679 ± 0.03	0.550	0.422	0.822 ± 0.08	0.637	0.302
Gradient boosting						
Trees						
5	0.678 ± 0.05	0.586	0.298	0.818 ± 0.09	0.571	0.402
10	0.663 ± 0.05	0.651	0.206	0.831 ± 0.05	0.56	0.403
25	0.668 ± 0.05	0.568	0.312	0.815 ± 0.08	0.663	0.396

#### Important predictors

In system B, we include historical positive and negative test rates, which provide another predictive feature that holds significant explanatory value in system B: the inclusion of test rates provides a marginal model improvement of $3.6\%$, over double the improvement of the next most valuable feature type, movement data (Fig. 3b). Wind direction and speed are important predictors, as is sow gestation feed and the number of incoming pigs (denoted by *Swine Movement (1st)* and *Swine Movement (2nd)*, which describe the number of incoming swine from the farms sending the first and second most swine, respectively (Supplementary Methods 3.2)). The incorporation of farm biosecurity data, unavailable in system A, also proves to be a useful factor: the manure storage method for PRRS, the employee access plan for PRRS and PEDV, and the carcass disposal method for PEDV and MHP. With the notable exception of test rates, the importance of various groups of features varies between diseases; in particular, MHP stands out as having important predictors that differ from the other diseases, with test rates and movement data providing outsized explanatory value in this case, and source distance data and biosecurity data not providing value. Diagnoses on source farms and feed data provide much higher explanatory value for IAV than for other diseases, where cull and mortality data provide more value than feed data. These results are available in Figs. 2b and 3b.

#### Performance with selected important predictors

In system B, model performance consistently peaks with less than ten features, after which overfitting leads to relatively steady decreases in performance across all models. In particular, models with six features provide consistent performance, with test set scores of 0.585, 0.587, 0.728 and 0.748 on PRRS, PEDV, IAV, and MHP, respectively. After six features, our model’s cross-validation scores decrease slowly, by $-0.13\%$ on average with each feature addition through ten features (Fig. 4).

## Discussion

The use of machine learning in veterinary epidemiology is still in its infancy, but it has the potential to significantly contribute to the field. This study presents an approach to infection prediction on swine farms through the development of machine learning models. Our approach shows a good prediction ability for some of the most important endemic pathogens in the swine industry.

Previous work in predicting outbreaks on swine farms using machine learning include a gradient boosting model of PEDV outbreaks on sow farms with near real-time prediction capabilities using five weeks of historical data and one week of predicted future data^7^, a model of PEDV outbreaks on sow farms using movement and local area spread data^29^, and a machine learning model of PRRS outbreaks on finishing farms^27^. As suggested in Paploski et al., we expand our survey of machine learning models to include neural network models in addition to the random forest and gradient boosting models used in these works^7^. We consider multiple diseases (PRRS, PEDV, IAV and MHP) in our analysis of one production system (system B) in contrast to the single-disease focus of these previous works, which focus on PEDV, PEDV, and PRRS, respectively. Our work considers production systems as a whole, including both sow and nursing/finishing farms in both systems. In addition, we present analysis and a comparison of two distinct farm production systems with differing levels of data availability, and show that machine learning is able to predict positive samples in both of these contexts.

The data used in this research was extracted from both internal swine farm collection and external sources, which allowed us to model various factors contributing to disease occurrence, including historical as well as current environmental, climatic and farm-level specific factors. We demonstrated the value of our approach under scenarios from two separate farm production systems with different data availability and quality, one with unbalanced and scarce data (system A) and one with a richer feature set and better data availability (sytem B). Due to a relative lack of data in system A, we group together all diseases to compile a dataset. Given that diseases often happen in tandem, with immune system weakness from one disease begetting another disease, this model still has high predictive value. However, the grouping of diseases may not be desirable if we want to inform target interventions focused to specific pathogens on farm, which is a limitation of this approach. The sample set in system A is quite unbalanced, with approximately 11% of samples being positive for all window lengths. This presents two challenges: models will be unbalanced toward the majority group (negative samples) and naïve accuracy metrics will report high scores even if predictions of positive samples are poor (for example, a model that predicts negative for any input, would obtain an accuracy of 89%)^30^. To address both of these challenges, we use balanced accuracy as the performance metric, with thresholds determined as the maximizer of the Youden’s J statistic on validation sets^31^. Balanced accuracy is the weighted accuracy of both positive and negative classes; thus, the same model that predicts negative for every input would only achieve a score of 50%. By selecting for this metric, we select for models that perform well on positive as well as negative samples.

The structure of the available data leads to test rates being an overly effective predictor in production system A that may not be applicable to other production systems or sample sets. This is a result of the fact that many farms have an overwhelming number of negative samples relative to their number of positive samples or vice versa. To illustrate this, we build a baseline heuristic model that solely utilizes historical test rates to predict outbreaks. On system A, this simple model is effective: it obtains a balanced accuracy of 0.883 on the test set with a 60-day window, surpassing all of the machine learning models. This observation is an artifact of the data: many samples exist on farms that have either a very high number of negative or positive samples relative to their total number of samples. The heuristic model achieves scores of 0.584, 0.578, 0.725, 0.752 on PRRS, PEDV, IAV and MHP in production system B, respectively, with the relatively high accuracy of IAV and MHP heuristic models corresponding with the endemic nature of these diseases^32,33^. As a result of this analysis, we discard test rates as a feature in system A but retain them in system B. Details on this heuristic model are available in Supplementary Discussion 2.3.

The inclusion of test rates in system B may contribute to models predicting continued infection, as historical rates of positive/negative tests are associated with samples being positive or negative. This effect is mild, however: the historical positivity rates are 1.66% and 1.59% averaged across all positive and negative samples, respectively, while the historical negativity rates are 3.80% and 5.90%, respectively.

In system A, the ensemble tree models outperform other models, with random forests and gradient boosting returning consistently high accuracies. These models are equipped to extract nonlinear signals from input features, with the ensembles providing a buffer against overfitting. In system B, the neural network model equipped with an auto-encoder architecture, such as that with layers of 32, 4, and 32 dimensions, generalizes well. These models are often close to our top performers on the cross-validation set but are not quite the best; however, they often produce the best results on the test set.

Permutation importance was used to find important predictors of positive samples, which does not depend on the type of machine learning model used^28^. We find that distance of nearby farms—a proxy for local farm density—total piglet inventory, the number of pigs weaned per mated female per year (PMFY), pregnancy rates and average gilt pool inventory (total gilt days per days in period) are all particularly valuable in distinguishing between positive and negative samples in the system A model. We note that close nearby farms implies high local farm density, and vice versa; our model shows that higher values of nearby farms (implying farms are farther away) is associated with less disease. Increases in PWMFY are associated with increases in positive samples, as are the average live born and pigs weaned per sow. Gilt pool inventory is likely associated as a predictive factor of disease because there is typically a correlation between disease and the frequency of gilt introductions to the herd: smaller gilt pool inventory and shorter total days probably indicates more frequent gilt introductions into the herd at an older age so there are more events to bring disease in and lower quarantine time as they are closer to breeding age.

In system B, test rates, wind direction and speed, sow gestation feed, and incoming swine movements are important predictors. Feed quantity likely shows up as a predictor due to its correlation with farm size, which can correlate with outbreaks; alternatively, it is possible that disease are transmitted through feed or contaminated feed delivery vehicles, in which case more feed deliveries leads to more opportunities for transmission. Specific features also hold particular value for specific diseases: movement data, for example, is valuable in the PEDV model, concurring with existing models that use movements to predict PEDV outbreaks^10^. Diagnoses of IAV on source farms is an important predictor for IAV. Biosecurity data proves important for PRRS, MHP and PEDV: the employee access point plan is valuable in PRRS and PEDV prediction, and the carcass disposal plan is valuable in PEDV and MHP prediction. Specifically, employee access plans that require showering in and out is associated with mild decreases in predicted infection probability for PEDV and mild increases for PRRS. This latter correlation is surprising but can be explained by the likely fact that showering is more frequently required on higher volume farms, which leads to additional opportunities for infection via feed delivery or swine movements. The rendering and biovator carcass disposal plans are associated with mild increases in predicted infection probability of PEDV and MHP, respectively. In addition, storing manure in a lagoon as opposed to a tank or deep pit is associated with predicted infection probability of PRRS.

The model in system A is a high-level model due to the grouping together of diseases. As such, this model models pathways concerning broad health issues; positive diagnoses, or predictions thereof, can reveal that there is a breakdown of overall health on a given farm. Given that diseases often happen in tandem, prompting a clinical practitioner to investigate holds value even at a high level. We contrast this with system B, where we train models on specific diseases. In doing so, we model at a lower level by analyzing epidemiological pathways.

We propose three main reasons for the relatively worse performance in system B versus system A. First, the distributions of positive and negative samples in test and training sets are highly reflective of each other in system A, with approximately 11% of both training and test sets being positive. This does not hold for all diseases in system B; while some diseases have train and test set distributions that reflect each other, others, such as PRRS and PEDV, do not. Specifics on these distributions are located in Supplementary Table  S5. This is unmitigable due to the time series nature of the data.

In addition, the distributions of sow and finishing farms are not reflective of each other between train and test sets for all diseases in system B. In particular, the diseases that have similar distributions of sow and finishing farms—PRRS and PEDV—do not have similar distributions of positive and negative samples. The percentage of sow farms in the training and test sets are located in Supplementary Table S6.

Finally, as movement data is not available before January 1, 2021, the majority of training samples do not have movement data. Of course, as the test set, consisting of the final quarter of samples, falls after this date, all of these samples do. As a result, our model infers movement trends from a small portion of training samples: 87.7%, 72.3%, 82.1% and 75.1% of training samples are lacking movement data for PRRS, PEDV, IAV, and MHP, respectively (Supplementary Table S7). The magnitude of the lack of movement data begs the natural question of whether this data is worth including whatsoever.

We retain movement data in system B for three reasons. One, to retain consistency with system A, whose samples all have movement and movement-derived features. Along these lines, the movement counts are not the only movement data-related features; we also derive the Source Distance, Source Test Rate, and Source Diagnostic features. Two, removing these features leads to worse model performance and generalization. Specifically, for our neural net model with 32, 4, and 32 neurons in three hidden layers, our cross-validation scores mildly improve without movement or movement-derived features, with balanced accuracies of $0.644 \pm 0.05$, $0.789 \pm 0.07$, $0.687 \pm 0.08$, and $0.727 \pm 0.15$ for PRRS, PEDV, IAV, and MHP, respectively. However, test set scores are 0.462, 0.542, 0.687, and 0.573, respectively, all worse than their counterpart with movement data. This suggests that movement data aids in generalization. Furthermore, we note that if movement data is not useful, this would be detected and this data removed in the feature selection process. The fact that movement and movement-derived figures are among the most important figures in Fig. 2 suggests this is not the case. Third, by illustrating the value of even a limited amount of movement data, we reveal potential avenues for performance improvement in the context of additional data.

These factors lead to distribution shift, where the features of the test set do not reflect those of the training set. Distribution shift is a known challenge in machine learning as it violates one of the major assumptions of machine learning models—that the samples on which they perform inference reflect, in distribution, the samples on which they were trained^34,35^. This is perhaps the major reason why the auto-encoder MLP performs most consistently on system B. By learning an efficient representation, it handles distribution shift well as it retains factors that shift minimally between training and validation sets. This confirms our intuition that the auto-encoder model is best able to separate input noise from true input signals compared to other machine learning models, an extension of this architecture’s known ability to denoise noisy versions of inputs^36^.

In this work, we consider all diagnoses as equivalent for both sample definition and feature extraction. In future work, it would be illuminating to determine the impacts of different types of diagnostic testing, particularly surveillance laboratory testing versus clinical-ordered laboratory testing. In the former, viral testing is performed on a regular basis, generally every two to four weeks, to detect illnesses before they are widespread, while in clinical-ordered testing, examinations are performed when there is evidence of an outbreak. Specifically, there is significant value in determining the level of efficacy of surveillance testing in predicting future disease especially in light of the non-trivial economic costs involved in testing. This can be done by training models on surveillance diagnoses and using them to predict, and by extension evaluate on, post-exposure diagnoses. Doing so, however, requires knowledge of whether diagnostics fall into the surveillance testing category or the post-exposure category, information our current dataset does not possess. If future datasets contain this information, performing this evaluation would provide significant value to the swine industry as a whole.

Our sow dataset is not annotated with whether samples are collected from sows or piglets. It is common for sows to test negative for diseases even as piglets test positive; in future work, it would be illuminating to model separate predictions for sows and piglets if such labels were available. In addition, vaccination may lead to positive diagnoses, particularly when live attenuated vaccines are used. The presence of vaccination data would allow for such diagnoses to be excluded, further improving models’ specificity.

## Conclusions

In this work, we developed ML models for positive infection prediction on swine farms. To address challenges with data availability and distribution shift, we combine our ML models with feature selection by permutation importance to feed our model the most important features. We show that this feature selection improves generalization. To further address the challenge of distribution shift, we employ an auto-encoder MLP model in the system B dataset, which is able to learn an efficient representation of the data and maintain good accuracy. Overall, we consider data from two separate farm production systems with low and high data granularity, respectively, and show that we can build models that adapt to cases with sparse data, where we infer health trends on farms, and cases with granular data, where we are able to predict infection for specific diseases.

We make five key contributions in this work. First, we show that production data and external factors, particularly weather, aid in infection prediction and are important tools to gauge the probably of having positive samples. Second, we are able to predict positive samples and provide positive sample probability at farm level on a daily basis. Third, we show that our model has the ability to provide advance warning both seven and 30 days in advance, a significant benefit in a situation where reaction time is critical in disease prevention and alleviation. Fourth, we analyze our results in the context of two farm systems, and show that our model is able to generalize to different scenarios of data granularity. Fifth, we show that data availability and distribution shift can significantly influence results, and propose methods of handling these challenges.

We achieve a balanced accuracy of 0.853 on the test set in system A with the top six features. In system B, we achieve balanced accuracies of 0.585, 0.587, 0.728 and 0.748 with the top six features on the test sets of PRRS, PEDV, IAV, and MHP, respectively, primarily due to distribution shift between the training and test sets. In addition, we are able to perform early prediction of infection, with good accuracy both seven and 30 days in advance. We show that production data, distances to nearest farms, and biosecurity data are important factors in predicting infections. In particular, the significance of biosecurity measures emphasizes the importance of taking such measures to prevent disease infection and by consequence outbreaks.

These approaches provide a strong baseline for future work in infection and outbreak prediction. Our next steps will be to integrate these approaches into user-friendly platforms such as the Disease BioPortal (https://bioportal.ucdavis.edu) to facilitate the use and interpretation of these models by veterinarians and other stakeholders. These models, combined with real-time data streams from swine farms, can be a powerful tool in infection and outbreak prediction and avoidance in the future at the farm level. In doing so, our approach can have a significant impact on both economic and environmental costs by lessening the spread and consequences of disease outbreaks in swine populations. The methods and findings of this study have significant implications for the swine industry and contribute to the growing body of research on disease prevention and control in animal agriculture using machine learning.

## Methods

### Data

#### Farm systems

Our analysis focuses on two swine production systems that we refer to as production systems A and B. Production system A centers on data collected between 2016 and 2021 on a farm system that consists of over 110 sites. For our uses, we consider farms that contain relevant diagnostic data relevant to infection detection; this leaves us with 60 total farms, of which 40 are finishing sites and 10 are sow sites. Production system B centers on data collected between 2017 and 2022. While this production system consists of 926 sites, we focus on the subset of 148 farms containing both production and matching diagnostic data. Of these, we match diagnostic data for 132 NurFin sites, 15 sow sites and 1 boar site, which we group together with the sow sites. Metadata on these production systems is available in Supplementary Table S13.

Data is provided in three types of records: diagnostic, movement, and production. Diagnostic data is collected by Polymerase Chain Reaction (PCR), enzyme-linked immunoassay (ELISA) and serology testing. Over 98% of the diagnostic data used is collected by PCR; a breakdown by disease is available in Supplementary Table S14. The diagnostic data used contains both surveillance testing and clinician-ordered testing (because of clinical signs of disease). This data is not labelled as being in such groups and as a result we group diagnostics together for evaluation. We note that endemic diseases may lay dormant and have outbreaks of infection at non-periodic intervals; detection of these positive samples is also valuable and included here. Many diagnostic samples are collected on regular intervals, with three, four, seven and twenty-eight day intervals between samples being routine in our dataset. However, irregular intervals are also common: there are 343 total unique intervals across all diseases and systems. Production data is split into two varieties: sow performance data and nursery/finishing performance data (close out). Details on these datasets are summarized in Table 3.

**Table 3:** Data summary.

	Production system A				
	Diagnostic	Movement	Production		Biosecurity policy
			Sow	Close out (finishing farms)	
Time granularity	Daily	Daily	Weekly	Production Cycle (105–250 Days)	N/A
Number of farms	60	60	10	40	
Number of features	1	1	102	70	
Date range	7/2014–11/2021	1/2016–11/2021	1/2016–10/2021	2/2016–8/2021	
Description	Diagnostic results of diseases tested at specific farm on given day	Logs of pig transfers between farm pairs on given day	Data collected on sow farms	Data collected on finishing farms	

	Production system B				
	Diagnostic	Movement	Production		Biosecurity policy
			Sow	NurFin	
Time granularity	Daily	Daily	Weekly	Weekly	Per Farm
Number of farms	926	896	16	132	736
Number of features	1	1	137	69	77
Date range	1/2018–7/2022	1/2021–7/2022	1/2017–7/2022	1/2017–7/2022	N/A
Description	Diagnostic results of diseases tested at specific farm on given day	Logs of pig transfers between farm pairs on given day	Data collected on sow farms	Data collected on nursing/finishing farms	Data describing farm management policies

We also collect meteorological data from a separate source, motivated by the knowledge that weather conditions can influence local area spread of disease^37^. We use the World Weather Online API to collect historical weather data. Specifically, we consider five meteorological features: maximum and minimum temperatures, average humidity, average wind speed, and average wind direction. Finally, we leverage biosecurity data for farms in system B: the estimated number of swine on site, the carcass disposal plan, the manure storage method, the employee access point plan (describing measures used to cross into the biosecure area), and whether there is a shared lagoon.

#### Sample definition

We now discuss the assumptions and methodology behind the samples we use for infection prediction. Specifically, we explore four main types of potential disease predictors:

1. **Direct contact predictors**, motivated by the assumption that the transportation of infected pigs would beget disease at farms accepting such pigs^8^. We model this disease pathway with three main types of features: movement quantity features, source distance features and source diagnosis features, which consist of the number of incoming pigs, the distances of farms sending pigs, and the diagnoses on the farms sending pigs, all during the historical window.
2. **Spatio-temporal predictors** attempt to model the local area spread of disease, potentially aided by weather^9,37^. These factors include the distances of the five nearest farms and five meteorological features: maximum and minimum temperature, humidity, wind direction, and wind speed.
3. **Historical predictors**, including historical production data, are prompted by the idea that past trends may lend insight into future trends. In system B, we focus on three subsets of the production features: mortality-related, feed related, and cull related. We also include historical testing rates, both on the farm of diagnosis and on its source farms (the former only in system B due to issues discussed in the *Discussion*).
4. **Farm-specific predictors** are static management attributes of a farm that may influence infection propagation, especially those related to biosecurity. This data is only available in system B. We focus on five main policy features: the estimated number of swine on site, the carcass disposal plan, the manure storage method, the employee access point plan, and whether there is a shared lagoon. Each of these features consists of multiple categories, with the exception of the estimated number of swine on site, which is quantitative. The categorical information is one-hot encoded to form numerical features which are concatenated with the rest of the feature set. The specific categorical features are available in Supplementary Methods 3.1.

Each predictor consists of a collection of features. Historical predictors and some direct contact predictors are compiled from the datasets enumerated previously; a detailed description of this is available in Supplementary Methods 3.1.

At a high level, we define a sample as follows. Suppose we have a diagnosis (negative or positive) at farm *i* on day *T*. We examine the features over a temporal window $W_{T, n}$ of *n* days ending on day $T-1$: $[T-n-1, T-1]$ and compile either their mean or sum over that time period for continuous and count-based features, respectively. Note that after normalization, these are equivalent. This leaves us with features that are a temporal snapshot of the various predictors over the *n* days preceding the diagnosis. As a result of the back-dating method used to define features, temporal windows may overlap. The only exception to this definition is our usage of close out data in system A, which is collected at a temporal granularity too low for consideration by a recent window. For these predictors, we use the close out data from the most recently completed production cycle as predictors as in Shamsabardeh et al.^27^. In combining sow and finishing farms into one sample set, there are production features that exist only in one farm type and are missing in the other (pregnancy rates on sow farms, for example). We impute using the mean of existing samples as necessary to unify our farm types into a joint sample set. We expect this imputation to have at most a minor effect on model performance as the imputation values retain the distribution of the original data to the maximal extent possible.

**Figure 5:**
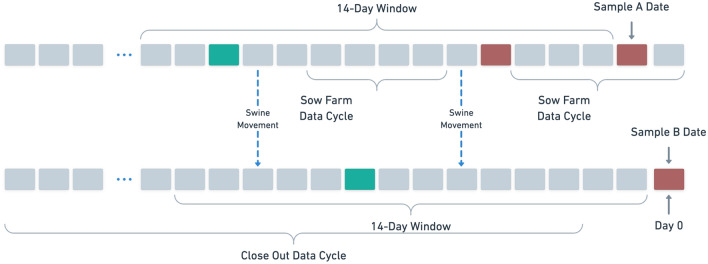
An example of the data collected for two samples using a 14 day window, with a Farm A at top and a Farm B at bottom. Green and red boxes denote days with a negative and positive diagnosis, respectively. We consider the data to be 0-indexed from the right so that samples A and B are taken on days $-1$ and 0, respectively.

A simplified example of sample definition is available in Fig. 5. Let the final day in Fig. 5 correspond to day 0; we have negative diagnoses occurring at Farms A, a sow farm, and B, a finishing farm, on day $-1$ and 0, respectively, from which we define one sample each. Farm A, a sow farm, includes the weighted average of the sow data collected during the current and previous cycle as well as the diagnoses at days $-5$ and $-13$. Farm B includes the diagnoses from Farm A as well as its own historical diagnosis at day $-9$. As Farm B is a finishing farm, we include the data from the close out cycle ending in day $-3$ in the sample.

#### Resultant sample set

Using the aforementioned sample scraping process, we build tabular sample sets for different window lengths: 14, 30, 60 and 90 days in system A. In system B, we focus on a 60 day window. As window lengths may extend past the first available day with data, some potential samples end up being discarded, leading to variations in the number of samples and slightly lower numbers of samples than there are diagnoses. Our final sample set for system A consists of 1014, 1007, 991, and 972 samples for windows of 14, 30, 60 and 90 days, respectively. In all cases the dataset is imbalanced in system A: the positive class is only 11.1%, 11.2%, 11.4% and 11.2% of all samples when $n=14,\; 30,\; 60\; {\text {and }} \; 90$, respectively. The number of features varies slightly, with longer window lengths having additional source features as movements have more opportunity to occur in longer windows. Additionally, the number of samples decreases with window length as only samples whose entire historical window is contained in the dataset are included.

Due to improved diagnostic data availability in system B, we are able to perform experiments for four respective diseases: PRRS, PEDV, IAV and MHP. Here, we focus on a window length of 60 days and obtain a sample set for system B consisting of 3215, 3366, 681, and 343 total samples for PRRS, PEDV, IAV and MHP, respectively, of which 708, 475, 271 and 97 are positive, respectively (Supplementary Tables S8 and S9). While the sample counts for some diseases—specifically, IAV and MHP—are relatively limited, we have a more balanced sample set overall than in system A. This balance, however, is not stable in time, with earlier samples—which compose the training set—having different distributions than the test set. As a result, our model maintains higher performance in system A than in system B, despite the latter appearing to have a more balanced dataset at first glance.

### Machine learning

We consider six machine learning models with different combinations of the features to determine the model that generalizes and performs best. Specifically, we consider logistic regression, support vector machines, decision trees, gradient boosting, and random forests in both systems; in system B, we also consider a neural network model. The model pipeline consists of five stages: standardization, feature selection, dimension reduction, model inference and threshold evaluation.

The standardization stage consists of subtracting each feature by its mean and then dividing by its standard deviation in order to make the features scale-invariant. In the feature selection stage, we select an optimal subset of features to maximize performance of the model. As a benchmark, we first analyze models with all features before performing a feature selection. Following this, we utilize a dimension reduction by principal component analysis (PCA) to reduce the dimension of the data to improve the model’s ability to generalize. In system B, we use a dimension reduction across all features. In system A, we explore both a standard dimension reduction as well as a stratified dimension reduction, wherein we perform two dimension reductions, one each for the sow and close out features, and then recombine them with the other features post-reduction. Due to the imbalance between classes in this binary classification, we select balanced accuracy as the metric to evaluate model performance. Additional details and motivation on these choices and stages are available in Supplementary Methods 3.3.

### Important predictors

We assess the relative importance of each feature to the highest-performing models—random forests in system A and MLPs in system B—via two approaches: a permutation feature importance and a forward feature importance, similar to Shapley values^38,39^. Note that these analyses do not precisely determine which features are the best predictors but rather which features are most important to a particular model; as the model is not perfect, feature importance is at best a biased estimator of the predictive value of particular features.

In the first case, the values of a single feature are randomly shuffled in the validation set and the model’s score is compared to its score pre-permutation; the difference is defined to be the permutation feature importance of the shuffled feature. This shuffling is repeated multiple times—in our case, five—for each feature. A higher score represents a higher contribution to the model from that feature: removing that feature provides a larger decrease in performance relative to others.

Permutation feature importance can fail to accurately analyze feature importance when features are highly correlated, either by assigning uniformly lower importance values to each member of a set of correlated features or by assigning group’s entire feature importance to one of the correlated features.

To address this limitation, we also consider a marginal feature importance, which proceeds as follows. We begin by training sets of models $M_1,..., M_m$ where the model set $M_i$ consists of a subset of models $$\{M_i^j\ | j = 1,..., \left( {\begin{array}{c}m\\ i\end{array}}\right)$$ trained on a feature set $X_i^j$, a combination of *i* features taken from the *m* total features.

Armed with these models, we can compute the performance gain provided by any given feature *a*: we simply determine the pairwise difference in the scores of models of size *i* such that $a \notin X_i^j$ with corresponding models of size $i+1$ that contain the same sets of features as those of size *i* but with the addition of *a*. The performance gain of feature *a* is the mean of all of these pairwise differences.

Consider the following simplified example. Suppose we train a model with three tabular features *a*, *b*, *c*. Then the performance gain provided by feature *a* is the mean of the scores of the set $\{M_{abc}-M_{ab}, M_{ab}-M_{b}, M_{ac}-M_c\}$. Performing this forward feature importance across model combinations requires training the model for every possible combination of features, which is computationally infeasible with large numbers of features. For example, in system A, with 219 features, this requires the training of $$\sum _{n=1}^{219}\left( {\begin{array}{c}219\\ n\end{array}}\right) \approx 8.425 \cdot 10^{65}$$ models. As a result, we split the features into their respective sources and perform this feature analysis across these smaller splits of feature groups.

### Performance with selected important predictors

Given the knowledge of feature importance, we retrain the model with selected sets of features to find the feature subset with the best predictive power. Specifically, we add an additional step to the pipeline before the dimension reduction in which we select a subset of features to pass to the remaining steps in the model. In order to select these features, we determine the permutation importance of the features through a cross-validation procedure utilizing a random forest classifier. This cross-validation is performed on every training fold of the overall five-fold cross validation. In other words, it is a cross-validation within a cross-validation.

### Early prediction

It is valuable to be able to detect warning signs of infection in the near or more distant future, so as to be able to prevent or mitigate the impacts of infections with sufficient lead time. To evaluate the model’s ability to perform future forecasting and provide infection predictions with lead times, we define two new sets of samples with lags of 7 and 30 days using data from system A. In other words, given a diagnosis on day *T* and a window of length *n*, the feature collection window $W_{T, n}$ is the time range $[T-n-l, T-l]$, where $l\in \{7, 30\}$ is the lag amount; we use a 60-day historical window here. This allows us to build a model to predict infection with 7- and 30-day advance warning.

### Supplementary Information

Supplementary Table S11.

Supplementary Table S12.

Supplementary Table S13.

Supplementary Table S14.

Supplementary Information 5.

## Data Availability

The data analyzed in this study is subject to the following licenses/restrictions: the dataset used in this study cannot be publicly available due to privacy agreements. Requests to access these datasets should be directed to Beatriz Martínez-López, beamartinezlopez@ucdavis.edu. The code used in this study can be made available upon request. Requests to access the code should be directed to Avishai Halev, ahalev@ucdavis.edu.
