# Supplementary material for: Infection prediction in swine populations with machine learning
Source: Sci Rep. 2023 Oct 18;13:17738. doi: 10.1038/s41598-023-43472-5 (PMC10584972; doi:10.1038/s41598-023-43472-5)
Supplement: Supplementary file 3 — Supplementary Table S13. [file 41598_2023_43472_MOESM3_ESM.docx]

**Supplementary Table S13.** Description of swine production systems.

|  | **System A (high-level)** | **System B (full)** |
| --- | --- | --- |
| **Breeding herds** |  |  |
| Herd size (# farms) | 39500 (9) | 282000 (65) |
| Herd Location (region) | South East | South Central |
| Production type | Farrow-to-wean | Farrow-to-wean |
| Gilt source/management | System Internal Multiplication- on and off site GDUs | Purchase isoweans/Off-site GDUs |
| PRRS status (MSHMP classification) | 3/4 | 1/2vx/2fvi/4 |
| *M. hyopneumoniae* status | Positive-Stable/Negative | Positive-Stable/Negative |
| PED status (MSHMP classification) | 4 | 1/2fvi/4 |
| IAV status | Positive | Positive |
|  |  |  |
|  |  |  |
| **Nurseries** |  | |
| Location (region) | South East | South Central |
| Weaned pig facility | -Wean-to-Market | Traditional nursery |
| Pig Flow | Multisource and Single Source all-in all-out (AIAO) | Multisource all-in all-out (AIAO) |
| Avg Capacity/per site (range) | 4800 (1000-16000) | 11196 (3500, 33856) |
|  |  |  |
|  |  |  |
| **Finishers:** |  |  |
| Location (region) | South East | South Central |
| Finishing facility | Wean-to-Market | Multisource grow-finish |
| Pig Flow | all-in all-out | all-in all-out |
| Avg Capacity/per site (range) | 4800 (1000-16000) | 8316 (2499, 21700) |
| **Testing modality**  **(Active surveillance)** | (frequency, number and type of samples, testing for what bug) Replacement Gilt Flow- 2x per turn, oral fluids- 4 pens sampled per 1200 head- PRRSv, SVA, PED, TGE, PDCoV  Suckling piglets on select sow farms- Processing fluids tested monthly from a sample of daily collections- PRRSv  Commercial Finishing- 1-2x per year- oral fluids from ~20% of sites- Lawsonia, PRRSv, PCV2, PED, TGE, PDCoV, SVA  Boar Stud- Every Collection (2x per week)- serum samples from 25% of boars- PRRSv | (frequency, number and type of samples)  Replacement gilt flow-weekly-6 oral fluids per 1000 head (PRRSV, PEDV/PDCoV/TGEV); monthly-30 tracheal swabs per 1000 head, pooled by 3 (MHP)  Suckling piglets all sow farms-weekly-3 processing fluids, pooled by 3 (PRRSV); monthly-3 processing fluids, pooled by 3 (PCV2/PCV3)  Due to wean sows from select sow farms-monthly-12 udder swabs, pooled by 3 (IAV-S)  Commercial Nursery-1x per turn-3 environmental swabs per 1000 head, pooled by 3 (PEDV/PDCoV/TGEV)  Commercial Finishing-1x per turn-1 environmental swab per 1000 head and 1 oral fluid per 1000 head (PEDV/PDCoV/TGEV) |
|  |  | Boar stud-every collection-15 blood swabs, pooled by 3 (PRRSV) and 1 environmental swab (PEDV/PDCoV/TGEV) |
|  |  |  |
