# Supplementary material for: Infection prediction in swine populations with machine learning
Source: Sci Rep. 2023 Oct 18;13:17738. doi: 10.1038/s41598-023-43472-5 (PMC10584972; doi:10.1038/s41598-023-43472-5)
Supplement: Supplementary file 5 — Supplementary Information 5. [file 41598_2023_43472_MOESM5_ESM.pdf]

Infection Prediction in Swine Populations with Machine Learning

Supplementary Materials

Avishai Halev, Beatriz Martínez-López, Maria Clavijo, Carlos Gonzalez-Crespo, Jeonghoon Kim, Chao Huang, Seth Krantz, Rebecca Robbins, and Xin Liu

1 Supplementary Results

Tables S1, S2 and S3 give the performance of additional machine learning models on both farm systems in comparison to the random forest, gradient boosting, and neural network (system B) models reported in the paper.

2 Supplementary Discussion

2.1 Dimension Reduction Analysis

Table S4 and Figure S1 are the results of our analysis of the model's dependence on the dimension reduction hyperparameter, the number of features selected for dimension reduction, in system A.

2.2 Distribution Shift

Tables S5 and S6 and detail distribution differences between the train and test sets. Table S7 details missing movement data in the training set in system B.

2.3 Test Rates and Heuristic Model

The structure of the available data leads to test rates being an overly effective predictor in the dataset in system A that would not generalize to other production systems or sample sets. This is a result of the fact that many farms have an overwhelming number of samples of one class and few or none of the other class.

To illustrate this, we build a baseline heuristic model based on the historical test rates. Specifically, consider a sample taken at farm i on day T that has historical positivity and negativity rates $P_T^{(i)}$ and $N_T^{(i)}$, respectively. Then we define the probability of infection at farm i on day T as

$$p(i,T) = \frac{P_T^{(i)}}{P_T^{(i)} + N_T^{(i)}}. \qquad (1)$$

On system A, this simple model is effective: with a 60-day window, it obtains a balanced accuracy of 0.883 on the test set, surpassing all of the machine learning models. This observation is an artifact of the data: many samples exist on farms that either have no or few tests of one class; with a 60-day window, a particularly extreme case consists of a farm with 577 negative samples – containing over half of the dataset – and no positive samples. The full distribution of samples by farm, the majority of samples occur at farms that only have negative samples, is available in Supplementary Figure S4. Ultimately, to maintain the robustness of the model, we remove historical rates from the feature set in system A.

The distributions in system B do not suffer to the same extent, with the heuristic model able to perform well in some cases but not all. The model achieves scores of 0.584, 0.578, 0.725, 0.752 on PRRS, PEDV, IAV and MHP, respectively. As a result, we retain test rates as a feature in this system.

3 Supplementary Methods

3.1 Data

This section contains additional details on the predictor categories.

Direct Contact Predictors

Direct contact predictors derive from the fact that the transportation of infected pigs is a major factor leading to infection and outbreak. These predictors are motivated by the idea that the movement of pigs between farms may beget disease at the farm receiving pigs; intuitively, infections are more likely to occur if diagnoses at the farm sending pigs imply previous disease infection or outbreak. We model this disease pathway with three main types of features: movement quantity features, source distance features and source diagnosis features, broken down below.

1. **Movement quantity features** enumerate the number of pigs sent to farm i within the window $W_{T,n}$ from each of its respective sourcing farms. The number of features is determined by the farm that has the highest number of unique source farms in its busiest window.

2. **Source distance** and **source diagnosis** features are compiled using the source farms from above. Source distance features are the distances of each of the respective source farms to the farm receiving pigs. Source diagnosis features, on the other hand, are the counts of positive and negative diagnoses at each source farm within $W_{T,n}$. For every source movement feature, there is one corresponding source distance feature and two source diagnosis features – one positive, one negative.

Spatio-temporal predictors

Spatio-temporal predictors attempt to model local area spread of disease between swine populations. We expect local area spread to occur more often between nearby farms; as a result, we add the distances of the five nearest farms to the farm in a given sample as features. In addition, climactic factors are known to affect disease transmission; consistent warm or cold temperatures, for example, often lead to faster spread of disease and outbreak intensity. To model this, we encode five meteorological features: maximum and minimum temperature, humidity, wind direction, and wind speed. Each feature is extracted daily over $W_{T,n}$ at which point their respective means are computed to give the final value.

Historical predictors

Our final set of predictors are historical predictors, which attempt to capture past farm performance that may correlate with infection and outbreak. Our main source of historical features are production data, which include a variety of recorded data: ranging from the number of litters per female to the total pigs born dead, and including statistics on feed consumption, pig weights, spending on veterinary services, among others.

In system B, we focus on three subsets of the production features: mortality-related, feed related, and cull related. We find that by training models solely with these features, combined with the other predictors, provides improved performance compared to using all production features.

Test Rates

Our final type of historical feature is historical test rates. Specifically, we define two features – the historical positivity rate and the historical negativity rate – as follows.

Let $P^{(i)}_{[0,T)}$ and $N^{(i)}_{[0,T)}$ be the number of positive and negative tests, respectively, at farm i across all days from the beginning of available data through day $T-1$. Then the historical positivity and negativity rates at farm i on day T are

$$P^{(i)}_{[0,T)}/T \text{ and } N^{(i)}_{[0,T)}/T, \tag{2}$$

respectively. We examine models with test rates for both the sample farm – at which the sample is defined – and its source farms. Due to the structure of the dataset, however, we remove test rates at the sample farm as features system A, while retaining them in system B. In both systems, we use the test rates at source farms as features.

Farm-specific predictors

Finally, we consider additional farm-specific predictors in system B. Specifically, we analyze biosecurity data, a type of data unavailable in system A. This data concerns the management policies of each farm. We focus on five

main policy features: the estimated number of swine on site, the carcass disposal plan, the manure storage method, the employee access point plan, and whether there is a shared lagoon. The estimated number of swine feature is continuous while the others are categorical; the categorical information is one-hot encoded to form numerical features which are concatenated with the rest of the feature set.

Biosecurity Features

The following is a list of the categories involved in the biosecurity features for system B. These features (with the exception of the estimation number of swine on site) are one-hot encoded and then appended with the numerical features. The estimated number of swine on site are appended as is.

1. Estimated Number of Animals (Swine) on Site
   - Quantitative, data redacted for privacy
2. Current Carcass Disposal Plan
   - Rendering
   - Burial On-Site
   - Incinerator
   - Compost
   - Biovator
   - Not Applicable/Data Missing
3. Manure Storage (method)
   - Lagoon
   - Tank
   - Deep Pit
   - Not Applicable/Data Missing
4. LOS Access Point Employees
   - Shower In/Out
   - Boot Change
   - Not Applicable/Data Missing
5. Shared Lagoon
   - Yes
   - No
   - Tank
   - Not Applicable/Data Missing

Resultant Sample Set

Tables S8 and S9 contain details of the sample sets used in the machine learning process.

3.2 Feature Names

This section describes the naming conventions behind the figures used in the text in Figures 1 and 2, and in Supplementary Table S9. All numbered features are zero-indexed.

- *Farm Density Indicator/Nearest Farm Distance (nth)*. These features are the distances in kilometers of the five nearest farms. They serve as a proxy for the local farm density: if the five closest farms are nearby and the distances are low, the local farm density is high, and vice versa.
- *Sow Production: <Feature Name>*. These are sow production features. Details on specific features, as well as a list of all features, are available in Supplementary Table S8.
- *Close Out: <Feature Name>*. These are key performance indicators on finishing farms in system A.
- *Nurfin: <Feature Name>*. These are production features on nursing/finishing farms in system B. Details on specific features, as well as a list of all features, are available in Supplementary Table S8.
- *Source Movement: nth*. This is the number of incoming swine from the nth source farm. Farm 0 indicates the farm sending the most pigs in the historical window and farm n denotes the farm sending the least pigs. The numbers $0, 1, ..., n$ corresponding to *Source Negativity Rate*, *Source Positivity Rate*, *Source Negative Tests*, *Source Positive Tests*, and *Source Distance* features, e.g. *Source Negativity Rate: 4* refers to the same farm as *Source Movement: 4* within a given sample. Note that farm 4 in one sample does not necessarily refer to farm 4 from another sample as the corresponding ordering of numbers of incoming pigs changes between destination farms and historical windows.
- *Source Positivity/Negativity Rate: n*. This is the historical positivity/negativity rate (days with positive/negative diagnoses/total days) on the nth source farm.
- *Source Positive/Negative Tests: nth*. This is the count of positive or negative tests within the historical window on the nth source farm. This differs from historical test rates in that test rates are normalized by the number of total historical days and are collected over all previous days, while positive/negative tests are strict counts and collected only over the historical window.

3.3 Model Stages

This section contains additional details on the model stages.

The first stage is standardization, where each feature has its respective mean subtracted and is then divided by its standard deviation in order to make the features scale-invariant. The motivation and process behind the four subsequent stages is discussed below.

Feature Selection

The next step is a feature selection, which attempts to select an optimal subset of features to maximize performance of the model. Feature selection differs from dimension reduction in that it is a supervised process; features are selected according to their ability to aid model performance. As this type of supervision can depend significantly on the performance of the model, we perform this step across multiple models to determine the best combination of features and model. As a benchmark, we first analyze models with all features before performing a feature selection; this can be thought of as an identity feature selection. We discuss feature selection methodology and results in depth in a separate section.

Dimension Reduction

Due to the relatively large number of features – ranging from 204 to 404 depending on the system, disease, and window – we reduce the dimension of the data to improve the model's ability to generalize. In system B, we use a dimension reduction across all features. In system A, we explore both a standard dimension reduction as well as a stratified dimension reduction, wherein we perform two dimension reductions, one each for the sow and close out features, and then recombine them with the other features post-reduction. This latter dimension reduction is motivated by the idea that linear correlations are likely to exist within the sow and close out data; running a dimension reduction separately on these datasets will best extract those relationships. In both cases, we use principal component analysis (PCA) to perform the dimension reduction; we choose the number of PCA components via the cross-validation process, discussed in the cross validation section.

Machine Learning Models

We examine five binary classifiers: logistic regression, support vector machines, decision trees, gradient boosting, and random forests. In system B, we also explore a multi-layer preceptron model. Specifically, we examine fully connected models and explore models with both two layers and three layers, the latter in an auto-encoder type architecture. We find that this architecture is particularly suited for system B, as the samples suffer from significant distribution drift. As this is less of a factor in system A, we do not report results with this model. The auto-encoder models are able to extract the most important signals, allowing for better performance on validation and testing sets. In all cases, we tune hyperparameters with the five-fold cross-validation procedure.

Metrics

Due to the imbalance between classes in this binary classification, we select **balanced accuracy** as the metric to evaluate model performance. Balanced accuracy can be viewed in two ways – first, it is the weighted accuracy of each class, so that performance on the smaller class is weighted equally to performance on the larger class:

$$\text{Balanced Accuracy} = \frac{\text{Correct predictions on true negatives}}{\text{Total true negatives}} + \frac{\text{Correct predictions on true positive}}{\text{Total true positives}}$$

Second, it is the average of the model's sensitivity and specificity:

$$\text{Balanced Accuracy} = \frac{1}{2}\left(\frac{TP}{TP+FN} + \frac{TN}{TN+FP}\right)$$

where TP, TN, FP, and FN are the true positive, true negative, false positive, and false negative rates, respectively.

While balanced accuracy scores avoid the optimism of receiver-operator characteristic area under curves (ROC-AUC) on imbalanced datasets, they do necessitate the choice of a specific threshold for prediction. As with our other hyperparameters, we determine the optimal threshold in the cross-validation process as the threshold that maximizes the Youden's J statistic on the validation set:

$$\begin{aligned} J &= \text{sensitivity} + \text{specificity} - 1 \\ &= \frac{TP}{TP+FN} + \frac{TN}{TN+FP} - 1; \end{aligned}$$

it is equivalent to the threshold that maximizes the difference between the true and false positive rates. Since these thresholds are chosen to maximize performance on the validation set under consideration, we evaluate the performance of the models with these thresholds on the test set.

Cross Validation

All of our hyperparameters are explored by cross-validation. We first perform a train/test split by setting the last quarter of the samples aside, to ensure that the model avoids time-series data leakage. With the remaining data, we perform a five-fold cross validation across all of our hyperparameters; in each case, the folds are split before any scaling/dimension reduction is performed to prevent data leakage. The full set of hyperparameters explored is in Table S10.

	Parameters	14 Day CV	14 Day Test	14 Day Thresh	30 Day CV	30 Day Test	30 Day Thresh
Random Forest	Trees						
	10	0.895±0.040	0.829	0.095	0.879±0.033	0.833	0.160
	25	0.892±0.037	0.878	0.190	0.873±0.027	0.828	0.198
	100	0.885±0.047	0.842	0.204	0.880±0.030	0.824	0.194
Gradient Boosting	Trees						
	10	0.890±0.020	0.849	0.064	0.891±0.025	0.801	0.053
	25	0.880±0.021	0.844	0.109	0.891±0.025	0.792	0.060
	100	0.888±0.012	0.840	0.073	0.889±0.028	0.801	0.064
KNN	Neighbors						
	5	0.805±0.047	0.741	0.2	0.828±0.037	0.694	0.28
	10	0.805±0.047	0.741	0.2	0.828±0.037	0.694	0.28
SVC	C						
	1	0.820±0.018	0.564	0.069	0.850±0.044	0.768	0.07
	0.5	0.820±0.018	0.771	0.073	0.851±0.043	0.777	0.069
Decision Tree	Criterion						
	Gini	0.750±0.051	0.784	1	0.761±0.075	0.766	1
	Log Loss	0.725±0.038	0.768	1	0.737±0.048	0.748	1
Logistic Regression		0.830±0.037	0.820	0.082	0.803±0.063	0.768	0.104
		60 Day CV	60 Day Test	60 Day Thresh	90 Day CV	90 Day Test	90 Day Thresh
Random Forest	Trees						
	10	0.918±0.022	0.789	0.212	0.898±0.025	0.882	0.216
	25	0.915±0.028	0.825	0.164	0.898±0.022	0.896	0.218
	100	0.913±0.026	0.792	0.212	0.894±0.026	0.891	0.186
Gradient Boosting	Trees						
	10	0.898±0.028	0.792	0.140	0.872±0.036	0.856	0.051
	25	0.898±0.028	0.758	0.141	0.860±0.038	0.868	0.062
	100	0.897±0.030	0.769	0.132	0.865±0.034	0.887	0.102
KNN	Neighbors						
	5	0.897±0.02	0.725	0.2	0.814±0.042	0.810	0.2
	10	0.897±0.02	0.725	0.2	0.814±0.042	0.810	0.2
SVC	C						
	1	0.894±0.021	0.716	0.069	0.861±0.051	0.898	0.081
	0.5	0.894±0.022	0.716	0.069	0.861±0.051	0.898	0.081
Decision Tree	Criterion						
	Gini	0.714±0.02	0.656	1	0.747±0.062	0.715	1
	Log Loss	0.701±0.016	0.633	1	0.739±0.053	0.699	1
Logistic Regression		0.849±0.043	0.694	0.14	0.771±0.063	0.891	0.092

Supplementary Table S1. Balanced accuracy with selected hyperparameters on system A. Balanced accuracy represents the average recall, weighted between positive and negative samples. Columns *CV* and *Test* correspond to balanced accuracy scores in cross-validation and on the test set, respectively, while *Thresh* gives the optimal threshold for that model as determined via the metric computation process. Higher thresholds imply better model discrimination between positive and negative predictions.

		PRRS			PEDV		
		CV	Test	Thresh.	CV	Test	Thresh.
MLP	Hidden Layers						
	(4, 2, 4)	0.637 ± 0.05	0.575	0.321	0.651 ± 0.14	0.537	0.688
	(32, 4, 32)	0.640 ± 0.06	0.523	0.256	0.765 ± 0.09	0.525	0.251
	(32, 32)	0.626 ± 0.06	0.487	0.183	0.760 ± 0.13	0.527	0.460
Random Forest	Trees						
	5	0.604 ± 0.06	0.618	0.480	0.697 ± 0.10	0.503	0.394
	10	0.619 ± 0.06	0.615	0.462	0.720 ± 0.11	0.511	0.354
	25	0.629 ± 0.05	0.564	0.468	0.723 ± 0.12	0.553	0.276
Gradient Boosting	Trees						
	5	0.635 ± 0.07	0.568	0.357	0.719 ± 0.09	0.567	0.144
	10	0.607 ± 0.06	0.614	0.430	0.679 ± 0.07	0.499	0.220
	25	0.632 ± 0.07	0.553	0.353	0.723 ± 0.10	0.554	0.164
KNN	Neighbors						
	5	0.570 ± 0.05	0.484	0.680	0.648 ± 0.10	0.515	0.800
	10	0.571 ± 0.05	0.475	0.560	0.646 ± 0.10	0.509	0.800
SVC	C						
	0.5	0.614 ± 0.08	0.603	0.336	0.726 ± 0.08	0.528	0.306
	1	0.614 ± 0.08	0.607	0.343	0.726 ± 0.08	0.528	0.304
Decision Tree	Criterion						
	Gini	0.540 ± 0.02	0.528	1.00	0.586 ± 0.06	0.500	1.00
	Log Loss	0.558 ± 0.02	0.566	1.00	0.632 ± 0.13	0.500	1.00
Logistic Regression		0.649 ± 0.06	0.507	0.294	0.715 ± 0.1	0.548	0.344

Supplementary Table S2. Balanced accuracy with selected hyperparameters on for PRRS and PEDV on system B. Columns *CV* and *Test* correspond to balanced accuracy scores in cross-validation and on the test set, respectively, while *Thresh* gives the optimal threshold for that model as determined via the metric-computation process.

		IAV			MHP		
		CV	Test	Thresh.	CV	Test	Thresh.
MLP	Hidden Layers						
	(4, 2, 4)	0.670 ± 0.04	0.637	0.430	0.723 ± 0.13	0.579	0.502
	(32, 4, 32)	0.661 ± 0.08	0.710	0.336	0.706 ± 0.16	0.641	0.343
	(32, 32)	0.654 ± 0.09	0.623	0.292	0.716 ± 0.16	0.633	0.445
Random Forest	Trees						
	5	0.669 ± 0.04	0.540	0.424	0.836 ± 0.07	0.589	0.282
	10	0.683 ± 0.05	0.560	0.462	0.82 ± 0.10	0.604	0.254
	25	0.679 ± 0.03	0.550	0.422	0.822 ± 0.08	0.637	0.302
Gradient Boosting	Trees						
	5	0.678 ± 0.05	0.586	0.298	0.818 ± 0.09	0.571	0.402
	10	0.663 ± 0.05	0.651	0.206	0.831 ± 0.05	0.56	0.403
	25	0.668 ± 0.05	0.568	0.312	0.815 ± 0.08	0.663	0.396
KNN	Neighbors						
	5	0.607 ± 0.10	0.655	0.480	0.740 ± 0.09	0.492	0.240
	10	0.600 ± 0.07	0.590	0.560	0.758 ± 0.08	0.545	0.240
SVC	C						
	0.5	0.664 ± 0.06	0.613	0.351	0.748 ± 0.18	0.489	0.319
	1	0.664 ± 0.06	0.608	0.341	0.822 ± 0.14	0.554	0.155
Decision Tree	Criterion						
	Gini	0.575 ± 0.07	0.500	1.00	0.774 ± 0.14	0.529	1.00
	Log Loss	0.556 ± 0.06	0.500	1.00	0.752 ± 0.11	0.546	1.00
Logistic Regression		0.713 ± 0.05	0.641	0.338	0.824 ± 0.06	0.718	0.35

Supplementary Table S3. Balanced accuracy with selected hyperparameters for IAV and MHP on system B. Columns *CV* and *Test* correspond to balanced accuracy scores in cross-validation and on the test set, respectively, while *Thresh* gives the optimal threshold for that model as determined via the metric-computation process.

	PCA Components	14 Day CV	14 Day Test	14 Day Thresh.	30 Day CV	30 Day Test	30 Day Thresh.
Random Forest	20	0.861±0.034	0.864	0.242	0.866±0.029	0.815	0.188
	50	0.873±0.042	0.858	0.216	0.864±0.041	0.768	0.194
	All	0.863±0.043	0.849	0.284	0.864±0.025	0.81	0.27
	10/10	**0.885±0.047**	0.842	0.204	**0.880±0.03**	0.824	0.194
	20/20	**0.885±0.036**	0.835	0.188	0.877±0.038	0.846	0.156
Gradient Boosting	20	0.849±0.037	0.855	0.093	0.851±0.032	0.817	0.02
	50	0.873±0.032	0.867	0.069	0.857±0.022	0.808	0.05
	All	0.865±0.049	0.885	0.046	0.863±0.034	0.819	0.146
	10/10	0.884±0.015	0.838	0.067	**0.891±0.024**	0.801	0.051
	20/20	**0.889±0.038**	0.851	0.056	0.881±0.022	0.81	0.044
		60 Day			90 Day		
Random Forest	20	0.907±0.02	0.763	0.226	0.871±0.044	0.889	0.166
	50	0.898±0.03	0.747	0.29	0.876±0.049	0.873	0.144
	All	0.899±0.04	0.743	0.364	0.892±0.034	0.866	0.276
	10/10	**0.927±0.027**	0.798	0.236	0.889±0.032	0.882	0.178
	20/20	0.910±0.029	0.783	0.272	**0.901±0.023**	0.868	0.146
Gradient Boosting	20	0.900±0.032	0.756	0.088	0.852±0.035	0.91	0.119
	50	0.877±0.031	0.749	0.056	0.853±0.027	0.887	0.068
	All	**0.904±0.027**	0.751	0.15	0.883±0.017	0.868	0.157
	10/10	0.901±0.033	0.785	0.121	0.865±0.037	0.898	0.047
	20/20	0.898±0.034	0.816	0.056	**0.869±0.032**	0.875	0.054

Supplementary Table S4. Effects of changing the number of components of the features set in system A. Components labeled as pairs are components of close out and sow features, respectively, while single values are components across the entire feature set.

	System B				System A
	PRRS	PEDV	MHP	IAV	
Train	0.271	0.135	0.226	0.367	0.114
Test	0.067	0.160	0.453	0.491	0.113

Supplementary Table S5. Percentage of positive samples in the training and test sets.

	System B				System A
	PRRS	PEDV	MHP	IAV	
Train	0.755	0.191	0.121	0.314	0.240
Test	0.764	0.080	0.035	0.678	0.250

Supplementary Table S6. Percentage of sow farms in the training and test sets.

System B				System A
PRRS	PEDV	MHP	IAV	
0.877	0.723	0.821	0.751	0.000

Supplementary Table S7. Percentage of samples in the training set that do not have movement data in system B.

Window	$n = 14$	$n = 30$	$n = 60$	$n = 90$
Features	204	213	219	220
+ samples	113	113	113	109
- samples	901	894	878	862
Total samples	1014	1007	991	972

Supplementary Table S8. Number of features and samples by window length in system A (all diseases). $+$ samples and $-$ samples denote positive and negative samples as defined by diagnoses on a given day, respectively.

	PRRS	PEDV	IAV	MHP
Features	273	405	273	259
+ Samples	708	475	271	97
- Samples	2507	2891	410	246
Total Samples	3215	3366	681	343

Supplementary Table S9. Number of features and samples by disease in system B. $+$ samples and $-$ samples denote positive and negative samples as defined by diagnoses on a given day, respectively.

Model stage	Hyperparameters
Dimension reduction	Stratified or unstratified<br>Component number
Feature selection	Number of features
Classification	Choice of classifier<br>Model-specific hyperparameters
Evaluation	Threshold values

Supplementary Table S10. Hyperparameters by model stage.

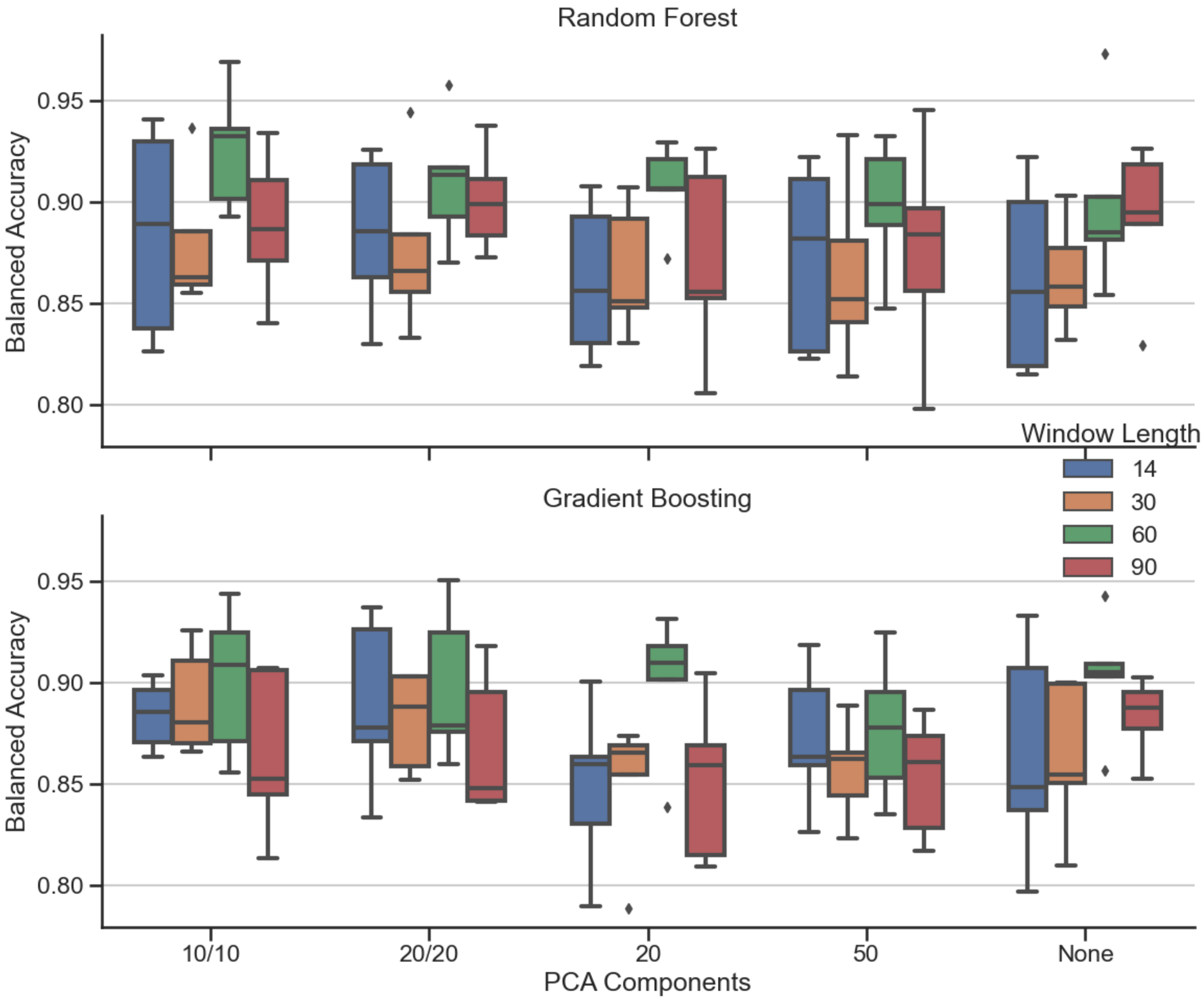

Supplementary Figure S1. Effects of changing the number of components of the features set across cross-validation splits on system A; points represent the scores on each split for random forest and gradient boosting models on the top and bottom, respectively.. Components labeled as pairs are components of close out and sow features, respectively, while single values are components across the entire feature set. None refers to no PCA, or usage of all features.

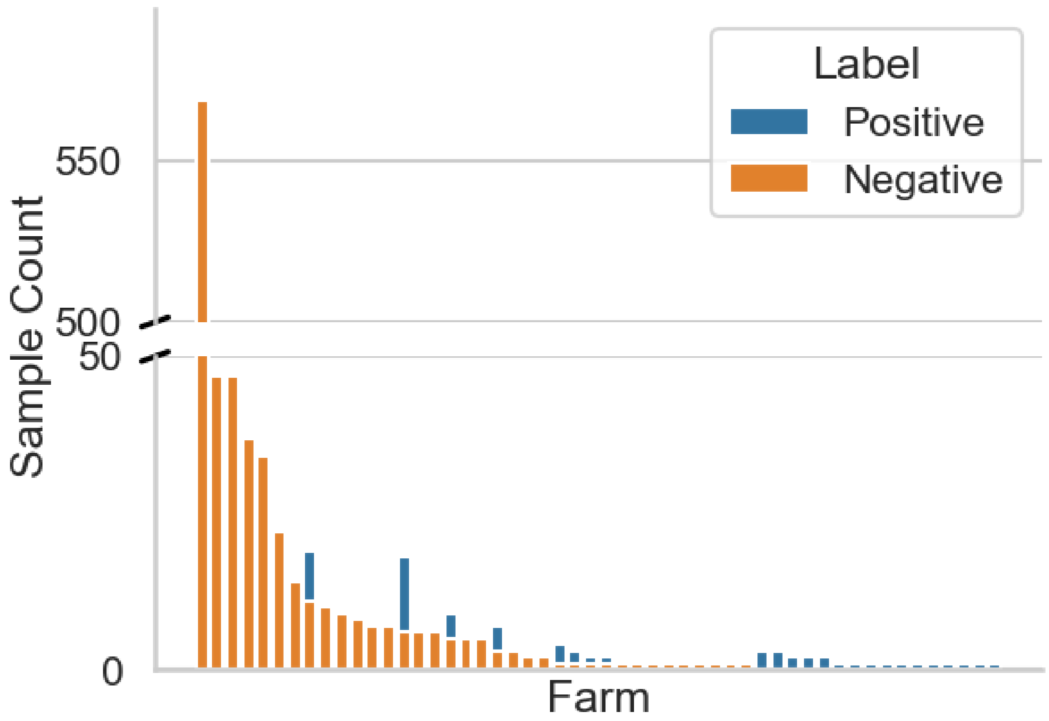

Supplementary Figure S2. Distribution of samples by farm in system A. Each bar represents a single farm.

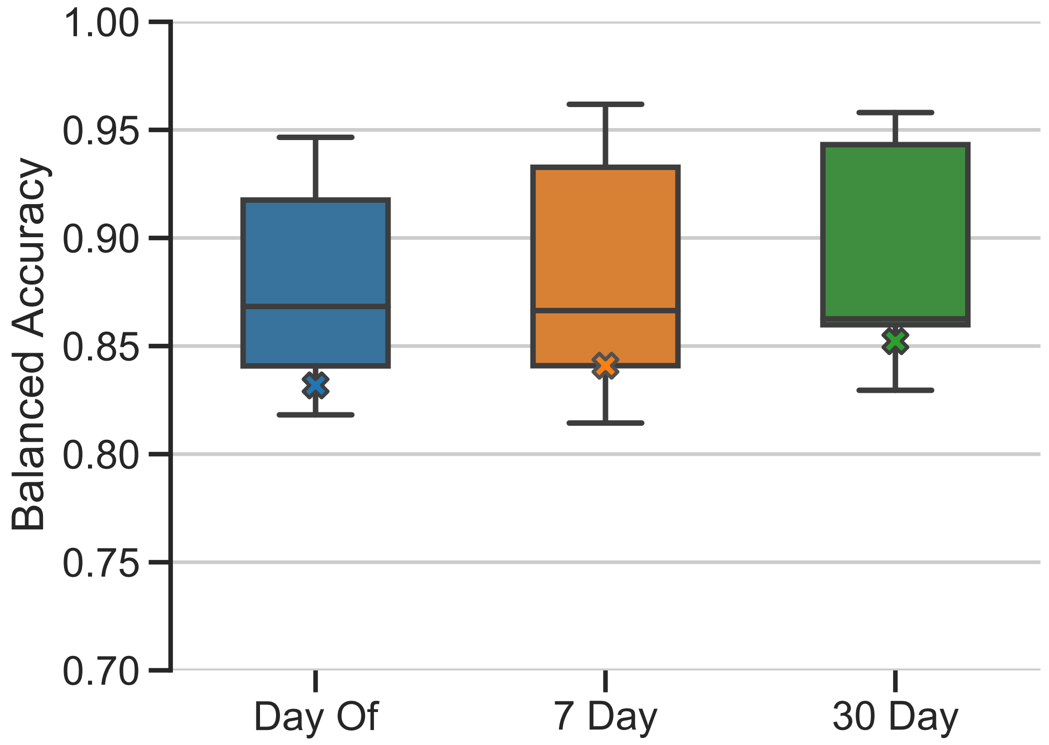

Supplementary Figure S3. Comparison of day-of prediction with lagged predictions on system A. X markers denote test set scores.

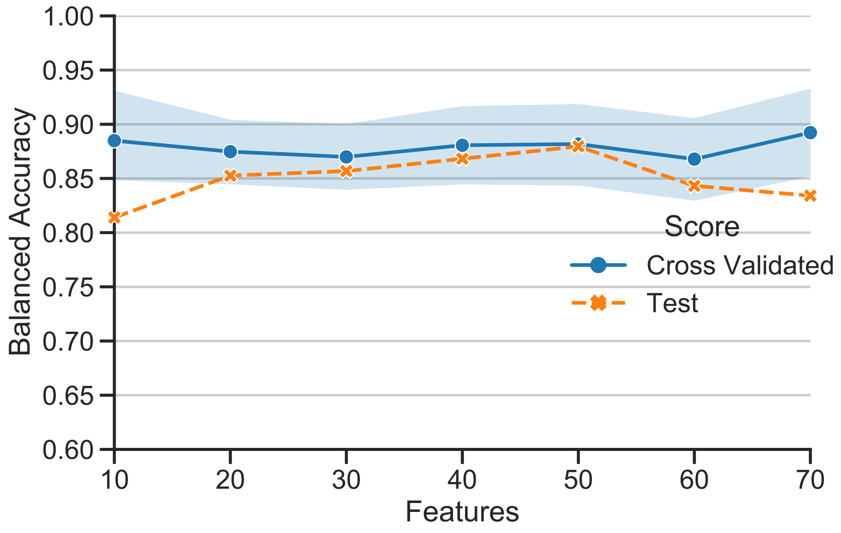

(a) System A

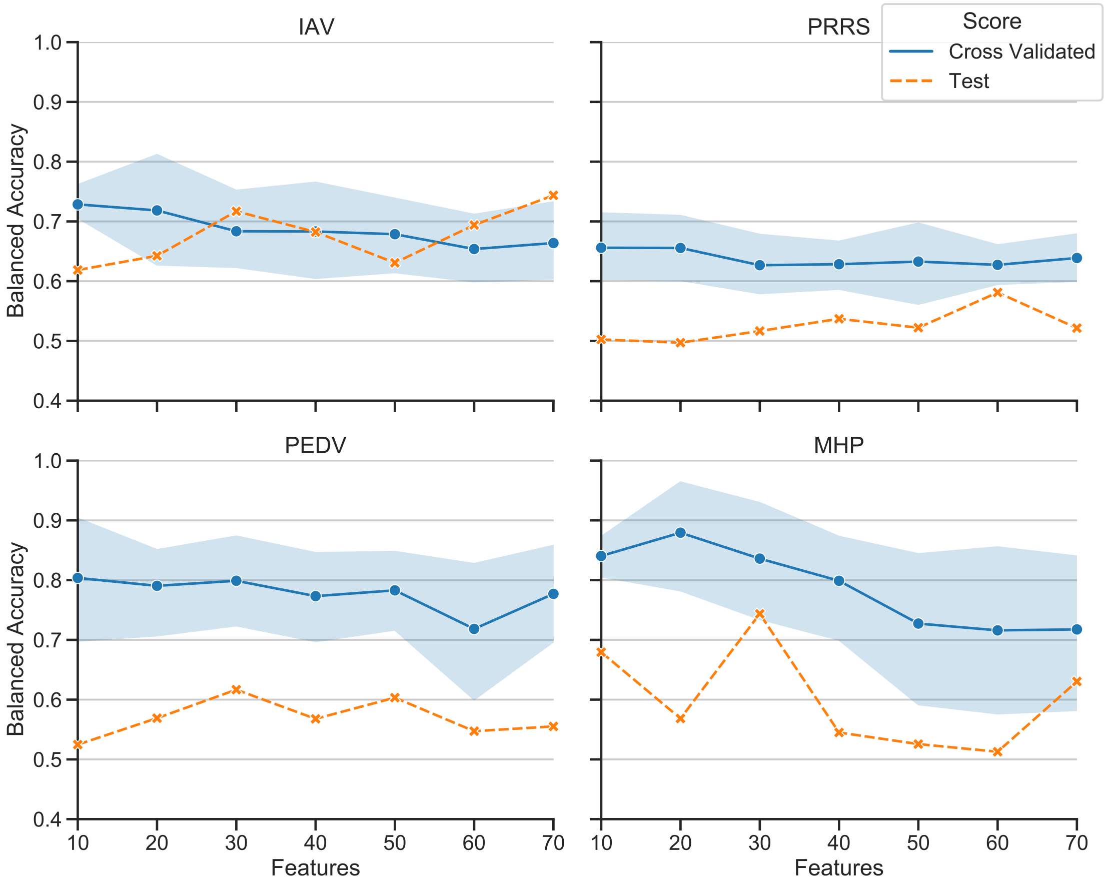

(b) System B

Supplementary Figure S4. Model performance with feature selection by permutation feature importance using all features, in increments of ten features.
